# Editorial: Mitochondrial dynamics and endothelial dysfunction: implications for metabolic disorders

**DOI:** 10.3389/fphys.2026.1829446

**Published:** 2026-03-27

**Authors:** Dorota Dymkowska, Aneta Balcerczyk

**Affiliations:** 1 Laboratory of Cellular Metabolism, Nencki Institute of Experimental Biology Polish Academy of Sciences, Warsaw, Poland; 2 Department of Oncobiology and Epigenetics, Faculty of Biology and Environmental Protection, University of Lodz, Lodz, Poland

**Keywords:** endothelial dysfunction, endothelial mitochondria, energy metabolism in endothelium, functioning of mitochondria in metabolic disorders, metabolic disorders, reactive oxygen species

Mitochondria are central regulators of cellular metabolism, redox homeostasis and signalling pathways that sustain vascular health. Although recognized for their role in ATP generation through oxidative phosphorylation, mitochondria have become increasingly clear as dynamic signalling hubs that integrate metabolic cues with cellular stress responses. In endothelial cells, mitochondrial function is essential for maintaining vascular tone, regulating oxidative balance, and supporting cellular adaptation to metabolic and environmental challenges (Dymkowska, 2021; Grossini et al., 2025; Singh et al., 2026). Disturbances in mitochondrial homeostasis, encompassing alterations in mitochondrial dynamics, impaired bioenergetics, defective mitophagy, and aberrant mitochondrial signalling, are now recognised as key contributors to endothelial dysfunction (Yu et al., 2012; Zhang et al., 2023). This dysfunction represents an early and pivotal event in the development of cardiovascular and metabolic diseases, including obesity and atherosclerosis (Casalena et al., 2020; Qu et al., 2022; Santos et al., 2023).

The Research Topic *“Mitochondrial Dynamics and Endothelial Dysfunction: Implications for Metabolic Disorders”* was conceived to highlight emerging insights into the complex interplay between mitochondrial biology and endothelial pathophysiology. The Research Topic brings together original articles, reviews and analytical studies that explore mitochondrial bioenergetics, mitochondrial quality control, metabolic signalling and systemic mediators influencing mitochondrial function. Together, these contributions underscore the growing recognition that endothelial mitochondria are not passive energy producers, but active participants in vascular signalling networks that shape metabolic health and disease.

Several articles in this Research Topic emphasise the importance of mitochondrial bioenergetics in cardiometabolic regulation. In the experimental study, using murine microvascular endothelial cells and cardiomyocytes Braczko et al. demonstrate that pharmacological modulation of cholesterol synthesis and turnover influences mitochondrial respiration and cellular energy balance. Their findings show that both atrovastatin and a synthetic PCSK9 inhibitor improve mitochondrial respiratory parameters and membrane potential under-mimicking conditions, with the PCSK9 inhibitor having particularly robust effects on mitochondrial function and cellular proliferation. These results extend the understanding of lipid therapies beyond their classical lipid-centric mechanisms and highlight potential mitochondrial contributions to their cardiovascular benefits.

Clinical insights into mitochondrial modulation in metabolic disease are provided by Satheesan et al., who investigated the effects of imeglimin (an antidiabetic agent) in patients with type 2 diabetes. Their prospective cohort study demonstrates that combination therapy involving imeglimin improves glycemic control while also increasing mitochondrial DNA copy number, suggesting enhancement of mitochondrial function. These findings align with growing evidence that pharmacological strategies targeting mitochondrial metabolism may provide therapeutic benefits in metabolic disorders characterised by impaired bioenergetics and oxidative stress.

As already mentioned, mitochondrial dysfunction also contributes to the pathogenesis of vascular complications associated with metabolic diseases. In the context of diabetic retinopathy Liu et al., employ bioinformatics to identify mitophagy-related biomarkers associated with proliferative diabetic retinopathy. Their study highlights key genes linked to mitochondrial quality control and immune infiltration, supporting the concept that dysregulated mitophagy contributes to retinal microvascular damage. Expanding this perspective beyond intracellular mechanisms, Zhu reviews the emerging role of exercise-induced circulating factors or exerkines in modulating retinal cell function and potentially mitigating diabetic retinopathy. These insights illustrate how systemic signals and lifestyle interventions may influence mitochondrial pathways and vascular health.

Beyond diabetes-associated complications, mitochondrial signalling also plays an important role in rare metabolic and genetic disorders. Yuan et al., provide evidence that circulating cell-free mitochondrial DNA levels are elevated in patients with Fabry disease and correlate with inflammatory cytokines. Their findings suggest that mitochondrial DNA released into the circulation may act as a damage-associated molecular signal that promotes inflammatory activation. Importantly, the study also proposes circulating mitochondrial DNA as a potential diagnostic biomarker, highlighting the broader clinical relevance of mitochondrial dysfunction beyond common metabolic diseases.

A growing body of work has also focused on the mechanisms linking mitochondrial dysfunction to insulin resistance and systemic metabolic dysregulation. In their comprehensive review, Feng et al., discuss how impairments in mitochondrial oxidative phosphorylation, excessive reactive oxygen species production and altered mitochondrial dynamics contribute to insulin resistance in type 2 diabetes. They highlight the role of microRNAs in modulating mitochondrial metabolism and redox signalling, emphasising emerging therapeutic opportunities that target the mitochondria-ROS-insulin resistance axis.

Metal homeostasis represents another layer of mitochondria regulation relevant to metabolic diseases. Wang et al., review the role of copper metabolism in diabetes and its complications, drawing attention to the recently described process of cuproptosis, a copper-dependent form of programmed cell death linked to mitochondrial metabolic pathways. Their analysis suggests that disturbances in copper homeostasis may amplify oxidative stress and inflammatory signalling, thereby contributing to diabetic pathology. Restoring copper balance could therefore represent a novel strategy for targeting mitochondrial dysfunction in metabolic disease.

Finally, Ding et al. provide a comprehensive bibliometric analysis of the rapidly expanding literature on mitophagy and atherosclerosis. This work maps the intellectual landscape of the field and identifies key molecular pathways, including NLRP3 inflammasome, NFκB signalling and mitochondrial DNA release, which link mitochondrial quality control with vascular inflammation. By highlighting current research hotspots and emerging themes, this study offers valuable guidance for future investigations into mitochondrial mechanisms underlying vascular disease.

Collectively, the articles in the Research Topic illustrate the diversity of mechanisms through which mitochondrial dysfunction contributes to endothelial pathology and metabolic disorders. They emphasise that mitochondrial processes are deeply connected with vascular health. Importantly, these studies also highlight the translational potential of targeting mitochondrial pathways through pharmacological agents, metabolic therapies and lifestyle interventions. Despite significant advances, many questions still remain unresolved. The precise mechanisms governing mitochondrial communication with other organelles, the role of extracellular mitochondria and mitochondrial DNA as signalling mediators, and the cell-specific contributions of mitochondrial dynamics within the vascular wall require further investigations.

In conclusion, the Research Topic reinforces the concept that mitochondria occupy a central position at the intersection of metabolism, inflammation, and vascular function. By integrating experimental, clinical and analytical perspectives, the collected articles expand understanding of how mitochondrial dynamics shape endothelial health and disease. Providing additional arguments of mitochondrial mechanisms may reveal innovative therapeutic strategies aimed at restoring vascular homeostasis and preventing cardiovascular complications.
